# Long-term association of a conditional cash transfer programme and maternal mortality in Ecuador: An ecological longitudinal study, 2010-2023

**DOI:** 10.1016/j.ssmph.2026.101963

**Published:** 2026-08-31

**Authors:** Sara Toros, Ronal Ruiz, Samuel A.G. da Silva, Pablo Álvarez, Monsermin Gualán, Klauss K.S. Garcia, Mauricio L. Barreto, Philip J. Cooper, Peter Craig, Natalia Romero-Sandoval, Julia M. Pescarini, Ana L. Moncayo, the Global Health Research Unit on the Social and Environmental Determinants of Health Inequalities (SEDHI) SEDHI members list

**Affiliations:** a Department of Infectious Disease Epidemiology & International Health, Faculty of Epidemiology and Population Health, London School of Hygiene & Tropical Medicine, London, United Kingdom; b Universidad Internacional del Ecuador UIDE, Quito, Ecuador; c Centro de Integração de Dados e Conhecimentos para Saúde (Cidacs), Instituto Gonçalo Moniz, Fundacao Oswaldo Cruz, Salvador, Brazil; d Grup's de Recerca d’Amèrica i Àfrica Llatines-GRAAL, Barcelona, Spain; e School of Health and Medical Sciences, City St George's University of London, London, United Kingdom; f Health Economics and Health Technology Assessment, School of Health and Wellbeing, University of Glasgow, Glasgow, United Kingdom; g Centro de Investigación para la Salud en América Latina (CISeAL), Pontificia Universidad Católica del Ecuador, Quito, Ecuador

## Abstract

• National longitudinal analysis of BDH and maternal mortality in Ecuador.
• No clear overall association between BDH coverage and maternal mortality.
• Stronger inverse associations in Afro-Ecuadorian and urban areas.
• Similar effects of the cash transfer were found before and during COVID-19 pandemic.
• Health-system capacity showed some evidence of modifying associations.

## 1 Introduction

Global maternal mortality in 2023 was estimated at 260,000 deaths, equivalent to one every 2 min (WHO, OECD & IBRD/The World Bank, 2025). Although global maternal mortality rates have fallen by about 40% since 2000, progress has slowed, and Latin America and the Caribbean (LAC) are among the few regions where maternal mortality has recently increased (WHO, OECD & IBRD/The World Bank, 2025). In LAC, most maternal deaths are related to preventable causes, primarily indirect conditions including noncommunicable and infectious diseases, followed by hypertensive disorders of pregnancy and haemorrhage, reflecting systemic failures in healthcare delivery (Cresswell et al., 2025).

Ecuador reflects these regional trends. According to World Bank model-based estimates, Ecuador's maternal mortality ratio (MMR) was 55 per 100,000 live births in 2023. Regardless of some progress in reducing maternal mortality, substantial geographic inequalities persist across the country (INEC, 2023; World Bank Group, 2023).These disparities occur within a health system that, despite constitutional recognition of health as a right and major reforms to expand universal coverage since 2008 (De Paepe et al., 2012), continues to face structural challenges, including uneven resource distribution, regional disparities in service quality, and shortages in skilled personnel particularly in underserved areas (De Paepe et al., 2012). Out-of-pocket expenditure still accounts for around one-third of total health spending in the country (WHO, 2014), creating financial barriers to antenatal, delivery, and emergency obstetric care (Jones et al., 2022). These inequalities disproportionately affect women in rural and low-income households, where most preventable maternal deaths occur (Mujica et al., 2023).

Addressing these disparities requires policies beyond the health sector. Poverty, education, and geographic isolation are key social determinants of maternal health (UNDP, 2015). Across LAC, social protection programmes, particularly conditional cash transfers (CCTs), have been widely implemented to reduce poverty and promote human capital by linking financial support to the uptake of essential health and education services (Fiszbein & Schady, 2009; Owusu-Addo et al., 2019). In many countries, CCTs have been associated with an increased utilisation of maternal health services (Glassman et al., 2013; Kusuma et al., 2016), but their effects on maternal mortality are less clear. Programmes such as ‘Prospera’ in Mexico (Parker et al., 2011) and ‘Bolsa Familia’ in Brazil (Rasella et al., 2021) suggest that CCTs may contribute to reductions in maternal mortality, although findings remain mixed and context-dependent (Lim et al., 2010; Powell-Jackson & Hanson, 2012).

In Ecuador, Bono de Desarrollo Humano (BDH) is the core component of the country's social protection system. BDH is a CCT that provides monthly cash transfers to low-income households, primarily mothers, conditional on the compliance with preventive health visits and children school attendance (Martinez et al., 2017). While the effects of the BDH programme on poverty reduction and under-5 mortality show some positive evidence (Pescarini et al., 2025), its impacts on other health and educational outcomes remain inconclusive (Pescarini et al., 2025), and no studies to date have examined its potential effect on maternal mortality.

**Fig. 1:**
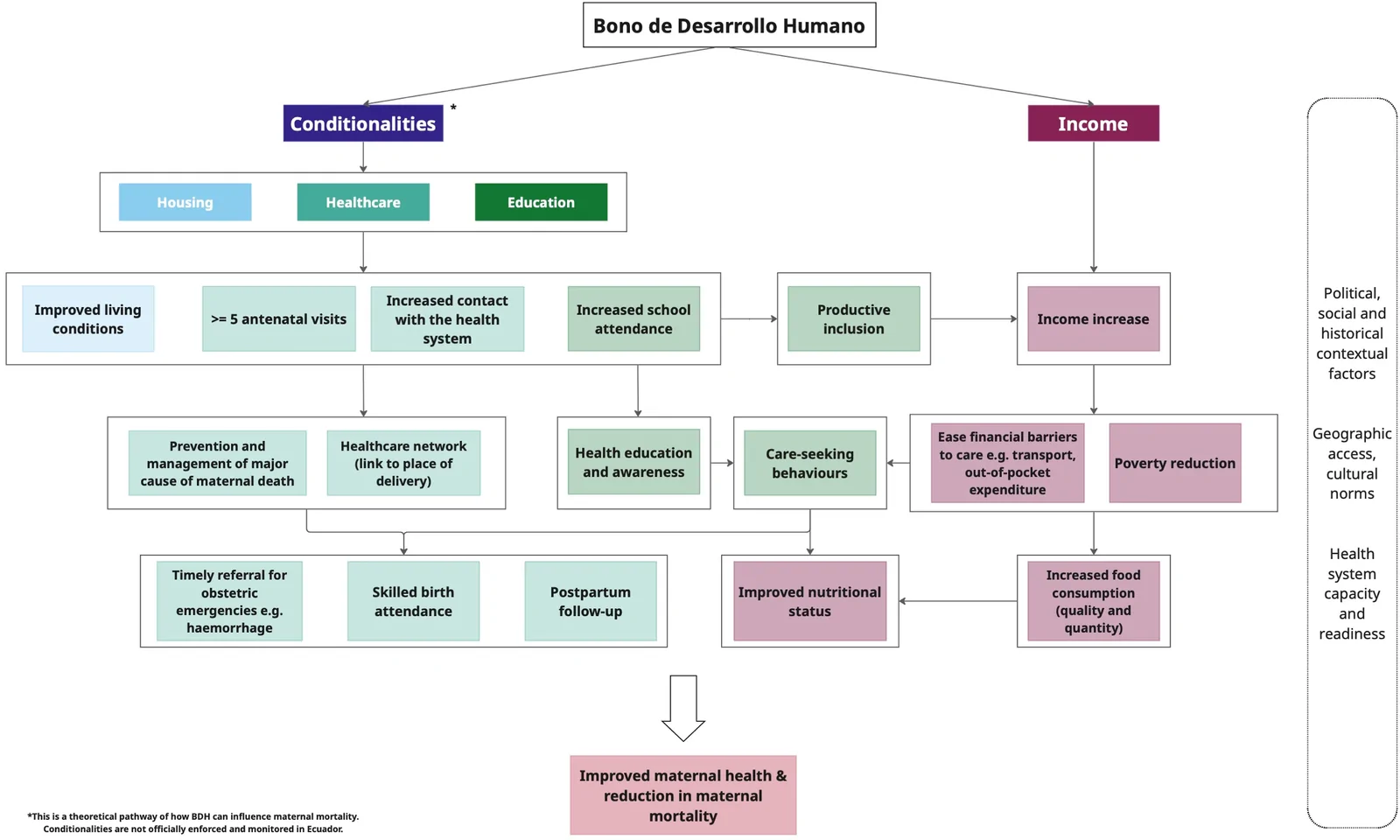
Programme theory of the effects of BDH on maternal mortality in Ecuador. Blue–green boxes represent pathways related to BDH conditionalities (healthcare, education, and housing) (UNDP, 2015). These are theoretical mechanisms, as conditionalities are not routinely enforced (Martinez et al., 2017). Pink–red boxes represent income-related pathways, through reduced financial barriers and improved nutrition. The right-hand column denotes broader contextual factors influencing these pathways.

This study aimed to evaluate the association between BDH coverage and maternal mortality in Ecuador at the canton level and to investigate differences in this relationship across socioeconomic and ethnic subgroups. Our hypothesis is that BDH acts on maternal mortality by increasing healthcare use, improving living conditions and nutrition, and reducing financial barriers to care, thereby promoting timely antenatal visits, skilled birth attendance, and overall maternal health (See Fig. 1).

## 2 Methods

### 2.1 Study design and study setting

This is an ecological longitudinal study using Ecuadorian ‘cantons’ (municipalities) as the units of analysis from January 2010 to December 2023.

Ecuador, an upper-middle-income country in northwestern South America with about 17 million inhabitants (INEC, 2023), is divided into 24 provinces, further divided into 221 cantons (second-level administrative divisions) (INEC, 2022). The country achieved notable social and economic gains during the 2000s, largely through public investment and social programmes, but progress has slowed since 2014 amid fiscal constraints and widening geographical and social inequalities (World Bank Group, 2019).

Ecuador implemented BDH in 2003 as a continuation of the Bono Solidario, an unconditional cash transfer implemented in the late 1990s (Martinez et al., 2017). BDH provides monthly cash transfers of US$50 to households in extreme poverty, with variable amounts up to US$150 depending on the number of minors (MIES). Payments are delivered in cash through more than 9000 authorised points. Beneficiaries are identified through the national Social Registry (Martinez et al., 2017; MIES), applying a welfare index based on household and socioeconomic characteristics. While eligibility requires compliance with co-responsibilities, including school attendance for children aged 5-17, at least five prenatal check-ups for pregnant women, and routine health visits for young children, compliance with conditionalities has not been systematically monitored and sanctions for non-compliance have rarely been applied (Martinez et al., 2017). At its peak, the programme reached over one million families and represented nearly 0.7% of GDP (World Bank Group, 2019), but the number of recipients declined by about 57% after a 2013 update that limited benefits to households in extreme poverty (Martinez et al., 2017; MIES). We reported a structured description of BDH using the Template for Intervention Description and Replication for PHP interventions (TIDieR-PHP) checklist (See Supplementary Table 1).

### 2.2 Data sources

We obtained data on BDH coverage, maternal mortality and key socioeconomic and demographic variables from publicly available Ecuadorian government databases at the cantonal level, the smallest administrative unit with information on BDH. Earlier years were not included, as information on BDH coverage was not yet available in public databases.

We extracted unidentified aggregated data on maternal deaths, live births, demographic socioeconomic data and health-system indicators from the National Institute of Statistics and Census (INEC), including the databases of births and deaths, the National Population Censuses (2010 and 2022) and Health Activities and Resources database (INEC, 2023); BDH coverage data from the Sistema del Registro Integrado de Programas Sociales (SIRIPS) (Gob E.C.). Data were combined into a panel-dataset by canton and year.

### 2.3 Variables

Following a previously validated criterion for assessing the quality of vital statistics in Ecuador the 221 cantons were classified into low-, intermediate-, and high-quality categories based on indicators of birth and death registration completeness and consistency (Moncayo et al., 2019). These indicators included the age-standardised mortality rate, the ratio between registered and estimated birth rates, the mean relative deviation of mortality and birth rates, and the proportion of ill-defined deaths. In this study, we used the resulting classification and restricted the primary analysis to 147 cantons with intermediate or high-quality data to minimise bias from under-registration and reporting errors in vital statistics; all cantons were included in a sensitivity analysis (See descriptive statistics in Supplementary Table 2).

The main outcome was the maternal mortality ratio (MMR), defined as the number of maternal deaths per 100,000 live births, calculated annually for each canton. Maternal deaths were identified according to WHO criteria and the International Classification of Diseases (ICD-10), with codes O00-O96, O98-O99 and A34, which include deaths during pregnancy or within 42 days of termination from causes related to or aggravated by pregnancy, while excluding accidental or incidental causes (WHO, 2019).

The primary exposure variable was BDH coverage at the cantonal level. Two indicators were created: (1) coverage of the eligible population (BDH-EP), calculated as the number of families enrolled in the BDH programme divided by the number of eligible families in the canton; and (2) coverage of the total canton population (BDH-TP), obtained by multiplying the number of beneficiary families by the average household size and dividing by total population. While the former captures programme coverage among eligible families, the latter reflects overall programme reach and potentially captures broader community-level effects, arising from the concentration of transfers within a locality.

Covariates included socioeconomic and health-system variables selected *a priori* based on previous literature and data availability: poverty quintiles (measured by the Unsatisfied Basic Needs Index (UBNI), a multidimensional indicator that captures deprivations in housing, education, basic services, and economic capacity) (INEC, 2021), illiteracy tertiles (rates among individuals aged 15 years and older), number of physicians per 10,000 residents, and number of hospital beds per 1000 residents. Annual values for the socioeconomic covariates were only available for 2010 and 2022, so the intervening years were estimated using linear interpolation and extrapolation, as in previous studies (see Technical Appendix in the Supplementary Material) (Moncayo et al., 2019; Rasella et al., 2021). Ethnicity and urbanicity data were only available from the 2022 Census. Cantons were classified according to the ethnic group(s) overrepresented relative to their national population proportion, resulting in five categories: Indigenous, Afro-Ecuadorian, Montubio, Mestizo/White, Mixed (cantons in which more than one ethnic group was overrepresented). Cantons were then classified into tertiles of urban population, corresponding to rural (lowest tertile), intermediate (middle tertile), and urban (highest tertile). Both were treated as time-invariant covariates.

### 2.4 Statistical methods

To assess the association between BDH coverage and MMR, we employed negative binomial (NB) regression models for panel data with canton and year fixed-effects (FE) specifications (cantons as units of analysis with repeated yearly observations from 2010 to 2023), using live births as an offset (see model equation):

$$\log\left({MD}_{it}\right)=\beta_{1}{BDH}_{it}+\beta_{2}X_{it}+\alpha_{i}+\gamma_{t}+\log\left({LB}_{it}\right)$$

$${MD}_{it}=maternal\;deaths\;in\;canton\;i,year\;t$$

$${BDH}_{it}=BDH\;coverage$$

$$X_{it}=adjustment\;variables\;\left(poverty,illiteracy,health\;system\right)$$

$$\alpha_{i}=canton\;fixed\;effects$$

$$\gamma_{t}=year\;fixed\;effects\;\log\left({LB}_{it}\right)=live\;births,included\;as\;an\;offset$$

The NB model was preferred over a standard Poisson regression as the outcome (maternal deaths per canton-year) exhibited over-dispersion, meaning the variance is greater than the mean (Hilbe, 2011). FE control for unobserved, time-invariant characteristics of each canton (such as geography, cultural factors, or historical differences) and account for the correlation of repeated measures over time, being more appropriate for evaluating intervention effects in panel data settings (Khandker et al., 2010; Wooldridge, 2005). Standard errors were clustered at the canton level to account for within-correlation over time.

We estimated rate ratios (RRs) and 95% confidence intervals (CIs) for the association between BDH coverage and MMR, both crude and adjusted for potential confounders, including poverty (i.e., UBNI), illiteracy, physician density, and hospital bed availability rate. Socioeconomic covariates were entered as categorical variables (quintiles or tertiles), while health-system indicators were treated as continuous.

To explore heterogeneity in association, interaction terms were introduced between BDH coverage and key stratifying canton-level variables: poverty, ethnicity, and urbanicity. We also tested potential changes in the association following major contextual events, including the 2013-14 update of the BDH eligibility criteria and the COVID-19 pandemic (2020–2021).

### 2.5 Sensitivity analyses

Sensitivity analyses were conducted to assess the robustness of the findings to alternative sample selection, model specifications, and analytical assumptions. These included analyses using different study populations (e.g., (1) including all cantons to test potential bias from canton exclusions, (2) restricting analyses to cantons with hospital beds and (3) at the province-level to assess the potential impact of geographic misclassification of maternal deaths, as women from cantons without hospital services may receive emergency obstetric care in neighbouring cantons, where deaths are subsequently recorded, or can be referred to province hospitals, where referral patterns are largely contained (Gob E.C., 2014)), and alternative modelling approaches (Poisson model). Full details and results of all sensitivity analyses are provided in the Supplementary Material.

All analyses were conducted in R (version 4.4.1). FE NB models were fitted using the *fenegbin()* function of the *fixest* package.

## 3 Results

**Fig. 2:**
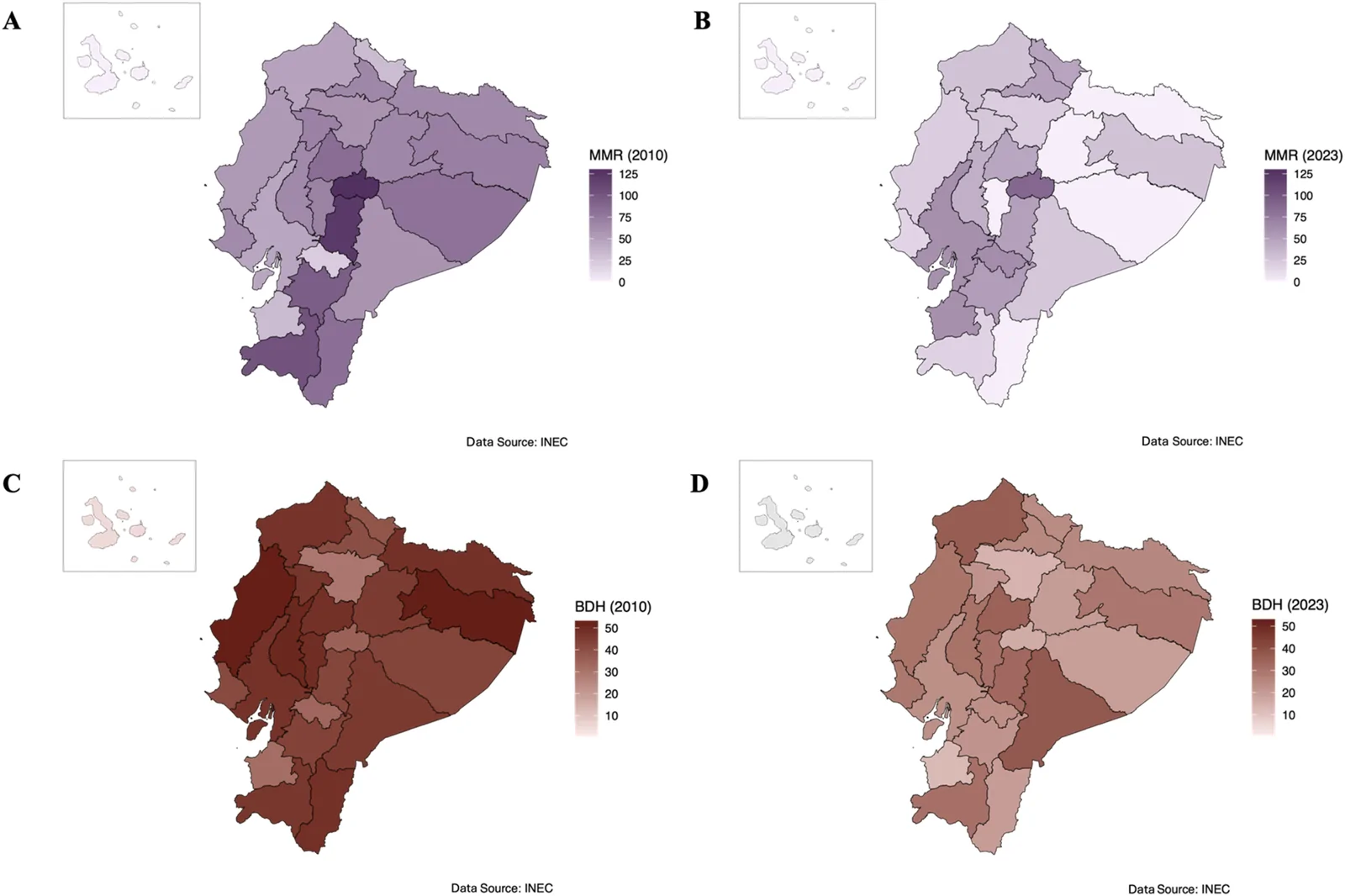
Maternal mortality ratio (MMR) per 100,000 live births and BDH coverage of total population by province, Ecuador, in 2010 (A, C) and 2023 (B, D). Inset (upper left): Galapagos Islands. Darker shades indicate higher MMR and BDH coverage. Data Source: INEC.

Between 2010 and 2023, 2142 maternal deaths and 4,013,271 live births were recorded in Ecuador, corresponding to a maternal mortality ratio (MMR) of 53.4 per 100,000 live births In 2010, provinces in the Andean region (Loja, Chimborazo and Tungurahua) had the highest MMRs, exceeding 100 deaths per 100,000 live births. By 2023, MMR was substantially lower in most provinces, although higher rates were observed in Guayas, El Oro and Cañar, and in Tungurahua (Fig. 2).

In 2010, BDH coverage of the total population was high and widely distributed, covering more than 40% of residents in most provinces. By 2023, BDH coverage had declined across much of the country, but remained comparatively high in provinces in the South-Eastern (Amazon) region, the Northern coastal region, and parts of the Andean region (Fig. 2).

Among the 147 of 221 cantons (66.5%) with intermediate or high quality of death and birth registration included in the main analysis, the mean MMR almost halved over the study period, falling from 41.4 to 21.4 per 100,000 live births (See Table 1). BDH coverage also declined, with BDH-TP falling from 42.3% to 24.1%, and BDH-EP falling from 60.5% to 49.1%. Over the same period, most socioeconomic indicators, such as the proportion of people in poverty (Unsatisfied Basic Needs Index), adult illiteracy, and physician density improved (See Table 1).

**Table 1:** Descriptive analysis of the selected study variables for specific years within the study period (N observations = 2058), 2010-2023, Ecuador.

	2010	2013	2016	2019	2023	Percentage change 2010-2023
Maternal mortality ratio (per 100,000 live births) and SD						
Overall	41.4 (92.2)	36.5 (84.9)	25.1 (65.2)	19.3 (65.7)	21.4 (58.8)	−48.2
Amazonica	36.1 (57.9)	38.7 (128.3)	20.9 (57.3)	NA	9.7 (32.1)	−73.2
Costa	39.2 (62.4)	27.5 (66.2)	22.3 (46.1)	14.5 (35.1)	20.3 (49.0)	−48.3
Sierra	44.6 (116.4)	44.5 (92.3)	28.6 (80.0)	26.8 (87.5)	24.5 (69.5)	−45.1

**BDH coverage**						
BDH coverage of the canton total population (%) (BDH-TP)	42.3 (14.2)	34.3 (13.1)	14.8 (10.4)	16.8 (11.4)	24.1 (11.3)	−43.0
BDH coverage of canton eligible population (poor households) (%) (BDH-EP)	60.5 (12.1)	53.6 (11.7)	23.8 (12.3)	29.6 (14.0)	49.1 (14.5)	−18.9

**Socioeconomic determinants of maternal mortality**						
Proportion of people in poverty (NBI) (%)	70.4 (14.3)	64.1 (14.6)	58.4 (15.2)	53.3 (15.9)	49.5 (17.0)	−29.7
Proportion of individuals older than 15 years who are illiterate	10.4 (5.6)	9.2 (5.2)	8.0 (4.9)	7.0 (4.6)	6.1 (4.3)	−41.4
Physicians rate (per 10,000 inhabitants)	9.6 (5.0)	13.0 (9.0)	16.1 (8.4)	17.7 (9.5)	22.5 (13.3)	134.5
Hospital bed rate (per 1000 inhabitants)	0.7 (0.8)	0.8 (0.9)	0.7 (0.8)	0.7 (0.8)	0.7 (0.8)	−2.42

Data is mean (Standard deviation (SD)). Maternal mortality is defined according to the International Classification of Diseases, 10th revision. Years were selected to illustrate changes, but data were available yearly for the entire study period. Insular data unavailable.

BDH = Bono de Desarrollo Humano.

**Fig. 3:**
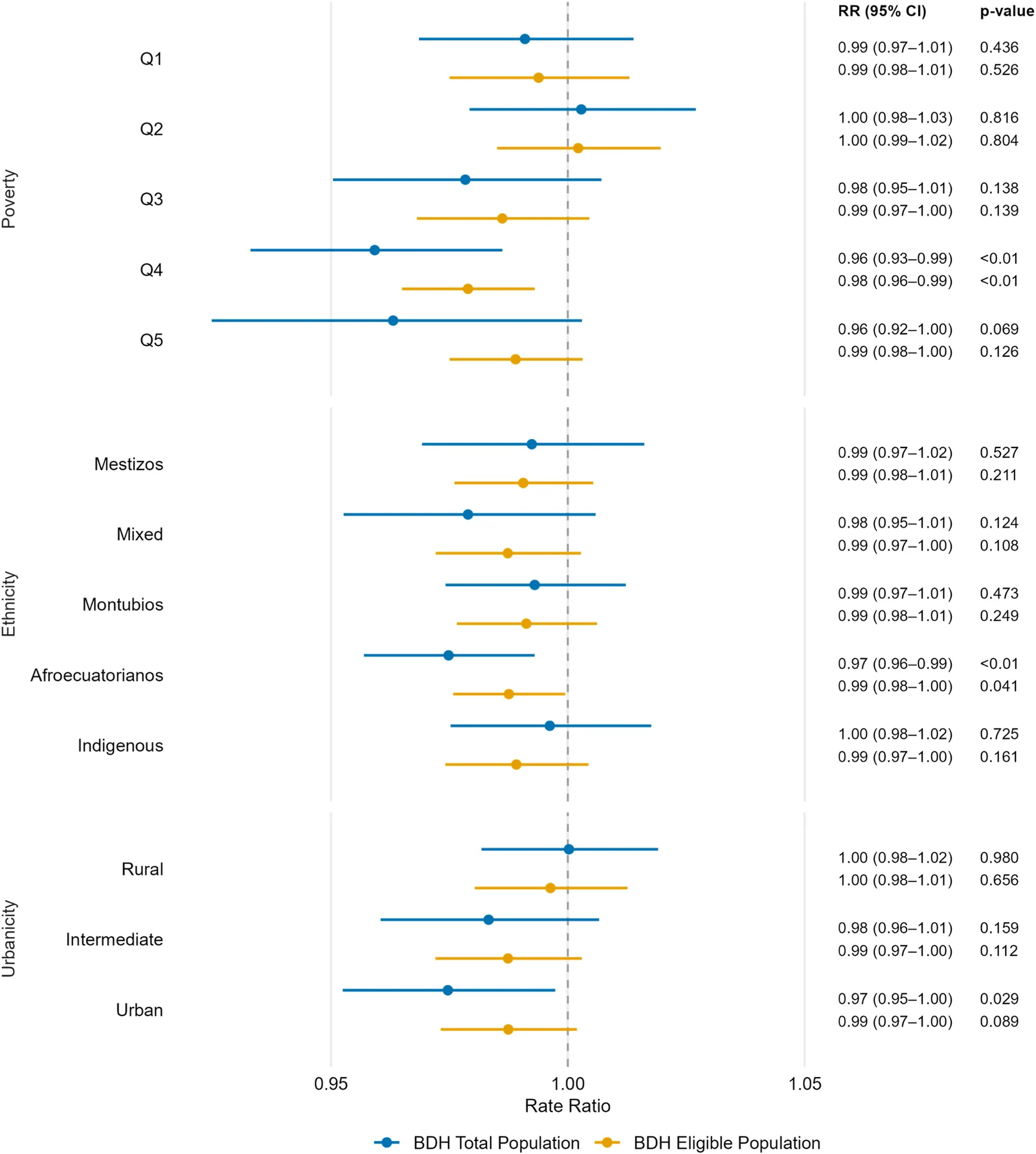
Adjusted rate ratio of the association between BDH and MMR, by poverty quintile (Q1 poorest, Q5 richest), ethnicity, urbanicity, across Ecuadorian cantons (cantons = 147, canton/year observations = 2058). RR = Rate Ratio; CI = Confidence Interval.

In models including only canton and year fixed effects, there was little evidence of an association between BDH coverage and maternal mortality (BDH-TP: RR = 1.00, 95% CI = 0.98-1.01; BDH-EP: RR = 0.99, 95% CI = 0.98-1.00). After adjustment for poverty, illiteracy, physician density and hospital bed availability, higher BDH coverage measures remained weakly associated with lower maternal mortality, with small effect sizes (BDH-TP: RR = 0.99; 95% CI = 0.97-1.00; BDH-EP: RR = 0.99; 95% CI 0.97-1.00). Across poverty quintiles, the association of both BDH-TP and BDH-EP and maternal mortality was close to one and CIs overlapped, indicating little evidence of effect modification (Fig. 3). The strongest inverse associations were observed in the fourth poverty quintile cantons (BDH-TP: RR = 0.96; 95% CI = 0.93-0.99; BDH-EP: RR = 0.98; 95% CI = 0.96-0.99). Across dominant ethnic groups, estimates were also generally close to the null (Fig. 3). An inverse association was observed in Afro-Ecuadorian-majority cantons (BDH-TP: RR = 0.97; 95% CI = 0.96-0.99), with smaller effect for BDH-EP (RR = 0.99; 95% CI = 0.98-1.00). When stratified by urbanicity, there was no evidence that BDH coverage was associated with MMR in rural or mixed rural/urban cantons. In predominantly urban cantons, BDH-TP showed a modest association (RR = 0.97; 95% CI = 0.95-1.00), while BDH-EP estimates were closer to null (RR = 0.99; 95% CI = 0.97-1.00). Corresponding rate ratios for a 10-percentage-point increase in BDH coverage are presented in Supplementary Table S11 to facilitate interpretation of effect size.

Before the 2013-14 eligibility update, there was little evidence of an association between BDH coverage and maternal mortality (BDH-TP: RR = 0.98; 95% CI = 0.97-1.00; BDH-EP: RR = 0.99; 95% CI = 0.98-1.00). In the post-update period, one-percentage-point increase in BDH coverage was associated with 3% reduction in MMR; 95% = CI 0.94-0.99), whereas the estimate for BDH-EP remained similar (see Table 2). During the COVID-19 pandemic (2020–2021), higher BDH coverage was associated with lower maternal mortality (BDH-TP: RR = 0.97, 95% CI = 0.96-0.99; BDH-EP: RR = 0.98, 95% CI = 0.97-0.99). Formal Wald interaction tests supported differences between pre- and post-update periods and between pandemic and non-pandemic periods for BDH-TP, but not for BDH-EP (see Table 2).

**Table 2:** Adjusted analysis of the association of BDH with MMR, Ecuador, stratified by eligibility update and pandemic years.

	BDH-TP	p-value^b^	BDH-EP	p-value^b^
	RR (95% CI)^a^		RR (95% CI)^a^	
**Eligibility update**				
Pre-2013	0.98 (0.97 – 1.00)	0.060	0.99 (0.98 – 1.00)	0.190
Post-2013	0.97 (0.94 – 0.99)	0.008	0.99 (0.97 – 1.00)	0.070
**Pandemic**				
Pre/post-COVID-19	0.99 (0.97 – 1.00)	0.370	0.99 (0.97 – 1.00)	0.350
COVID-19	0.97 (0.96 – 0.99)	0.010	0.98 (0.97 – 0.99)	0.005

p-value of interaction with eligibility update: BDH-TP = 0.01; BDH-EP = 0.39.

p-value of interaction with Covid-19: BDH-TP = 0.03; BDH-EP = 0.13.

RR = Rate Ratio; CI = Confidence Interval.

a Estimated using negative binomial regression for panel data with fixed effects, and adjusted for poverty, illiteracy, physicians' rate and hospital beds rate.

b Estimated using the Wald test for association.

Sensitivity analyses produced results consistent with the main models (Supplementary Table 3–10). In all specifications, RRs for BDH-TP and BDH-EP were close to the null and generally consistent with small reductions in MMR, supporting the robustness of the main findings.

## 4 Discussion

This study provides a long-term, aggregate-canton level evaluation of Ecuador's BDH and its association with maternal mortality between 2010 and 2023. Overall, BDH coverage is higher in structurally disadvantaged cantons, reflecting effective targeting of the programme. After adjusting for socioeconomic and health-system variables, BDH coverage was not associated with meaningful changes in maternal mortality over time. Our stratified analyses by levels of poverty, ethnicity and urbanicity indicate that any protective association of BDH is small but relatively similar across subgroups, with some suggestion of reductions in maternal mortality in urban and Afro-Ecuadorian-majority cantons and in cantons in the upper-middle poverty quintile.

World Bank estimates suggest that Ecuador's MMR was around 80 deaths per 100,000 live births at the start of the study period (World Bank Group, 2023), above the Sustainable Development Goal target of fewer than 70 per 100,000 live births (WHO, OECD & IBRD/The World Bank, 2025). Although national statistics used in our analysis are lower (approximately 60 per 100,000) (INEC, 2023), both figures indicate substantial preventable mortality and limited progress over time.

Our study found higher BDH coverage in poorer and otherwise disadvantaged cantons, consistent with earlier evidence that the programme effectively targets households with greater health needs (Poirier, 2020). Importantly, this level of equity was achieved without geographically restricting coverage, suggesting that nationwide implementation combined with strong eligibility criteria can effectively prioritise the poorest (Poirier, 2020). However, these high-coverage cantons continue to face substantial structural barriers to maternal health that contribute to higher MMRs. Spatial patterns were complex: although high mortality in poorer, Indigenous- and Afro-Ecuadorian-majority cantons aligned with longstanding inequities (Mesenburg et al., 2018; Rios-Quituizaca et al., 2022), high MMRs were also observed in some urban and better-resourced cantons. This may be partly reflecting the registration of deaths by place of occurrence rather than residence, as women from rural or poorly resourced cantons often travel to hospitals in larger cantons for care, particularly when specialised childbirth services are required, and these referrals often involve more complex cases with a higher risk of death. Among the 147 cantons analysed, 44 had no hospital beds throughout the study period, and 18 had only intermittent availability, with rural cantons disproportionately affected (39% versus 23% of urban cantons). Physician density increased from 9.6 to 22.5 per 10,000 population over the study period, yet specialists remained concentrated in Quito, Guayaquil, and Cuenca (Rodriguez et al., 2022), illustrating persistent inequities in availability of healthcare professionals.

The fixed-effects model without additional covariates provides an estimate closer to the total association between BDH coverage and maternal mortality. Because changes in poverty and education may partly lie on the pathway through which BDH influences maternal health, adjustment for these variables could attenuate the estimated association. However, the similarity between the crude and adjusted estimates suggests that adjustment had little influence on our findings. After adjustment for socioeconomic and health-system factors, canton-level and year fixed effects, associations between BDH coverage and maternal mortality remained small and close to the null. While our study found little evidence of an overall association, BDH's design includes two main plausible pathways through which it could influence maternal mortality (summarised in Fig. 1). First, through programme conditionalities, the requirement of at least five antenatal visits could support early detection and management of complications like hypertensive disorders, infections, and other pre-existing conditions, which were among the leading reported causes of maternal death in Ecuador in 2021 (Gob E.C., 2021). Regular antenatal care also facilitates counselling on birth preparedness and timely referral for obstetric emergencies. Consistent with this pathway, evaluations of Brazil's ‘Bolsa Família’ programme have shown reductions in the proportion of women with no prenatal care, increases in hospital deliveries, and lower delivery-related case fatality, supporting the relevance of these intermediate outcomes for improved maternal outcomes (Rasella et al., 2021). Second, through income support, BDH may ease financial barriers to care, improve nutrition, and increase health awareness, thereby fostering earlier care-seeking behaviours. However, in practice, BDH conditionalities have lacked strict enforcement, and no systematic mechanism exists to monitor compliance (Martinez et al., 2017), likely weakening these pathways and limiting observable reductions in maternal mortality.

Our findings contrast with those from Mexico and Brazil (Parker et al., 2011; Rasella et al., 2021), where CCTPs were associated with reductions in maternal deaths. In Brazil, the CCTP ‘Bolsa Família’ is implemented alongside a consolidated primary health care strategy, the Family Health Programme, considered one of the most effective in LAC (Macinko & Harris, 2015). This programme is responsible for monitoring health conditionalities, including antenatal care attendance, and has shown synergistic effects with cash transfers (Guanais, 2013). In Mexico, sustained increases in demand generated by the CCTP ‘Prospera’ have been linked to subsequent improvements in the supply of health services (Parker et al., 2011). Our findings align more closely with null associations reported in India, where limited results were attributed to inadequate targeting of the poorest women and gaps in the quality of obstetric care in health facilities (Lim et al., 2010). Although BDH effectively reached poorer and more structurally disadvantaged cantons, the absence of systematic monitoring of programme conditionalities may have weakened the pathways through which the programme could influence maternal health. Taken together, these comparisons suggest that programme design, implementation, and broader health-system factors are likely to influence the effectiveness of cash transfers, even when programme targeting is successful.

Subgroup analyses largely reflected the main results, with most estimates near the null and showing little evidence of heterogeneity across strata. Slightly stronger associations in urban cantons and those in the fourth poverty quintile may indicate settings in which service availability enables increased demand generated by cash transfers to translate more effectively into improved maternal outcomes. This aligns with evidence that CCTPs tend to have greater impact where health-system capacity is sufficient to absorb additional care-seeking (Ibarrarán et al., 2017). We explored whether local health-system capacity modified the association between BDH coverage and maternal mortality, evidence of effect modification was mixed (Table S12). Associations varied according to hospital bed availability, with the strongest inverse associations observed in cantons with the lowest availability of hospital beds, whereas little or no consistent variation was observed across physician density tertiles. These findings did not support our initial hypothesis that greater health-system capacity would strengthen the association between BDH and maternal mortality. One possible explanation is that physician density and hospital bed availability are imperfect proxies for the aspects of health-system performance most relevant to maternal survival, such as the quality of obstetric care, geographic accessibility, emergency referral systems, continuity of care and the availability of skilled birth attendants, for which comparable cantonal data were unavailable. An alternative, not mutually exclusive possibility is that BDH indirectly improves maternal death detection and reporting in poorer or high-coverage areas, as observed in other settings (Pescarini et al., 2022), potentially masking programme effects.

By ethnicity, protective associations were stronger in Afro-Ecuadorian-majority cantons. Given the long-standing inequities affecting these populations, including lower coverage of antenatal care, skilled birth attendance, and contraception (Mesenburg et al., 2018; Rios-Quituizaca et al., 2022), this pattern may suggest that BDH helps mitigate some disadvantages faced by historically marginalised groups. This interpretation should also consider the well-documented role of Afro-Ecuadorian women as primary caregivers within their households and communities, a responsibility they have historically assumed and which may influence health-seeking behaviours and programme uptake (Siguenza-Orellana et al., 2024). Nevertheless, maternal mortality remained high in both Afro-Ecuadorian- and Indigenous-majority cantons throughout the study period, indicating that BDH alone may be insufficient to address deep-rooted inequities shaped by colonial legacies, racism, and geographic marginalisation (Mesenburg et al., 2018). Indigenous women, in particular, face intersecting disadvantages (higher poverty, lower education, and limited access to essential services including clean water and sanitation), which increase health risks across the life course (Rios-Quituizaca et al., 2022). Integrating culturally sensitive and community-led strategies within BDH-linked health services, including health promotion initiatives, could further improve access and outcomes in vulnerable communities.

The COVID-19 pandemic created exceptional strain on health systems across Latin America, which accounted for 15% of global excess deaths in 2020-2021 despite comprising only 8.5% of the world's population (OECD/The World Bank, 2023). Ecuador experienced one of the region's highest levels of excess mortality, with more than 27% of households reporting barriers to accessing care (OECD/The World Bank, 2023): prenatal, delivery and postnatal care fell by 46%, 28%, and 38%, respectively (Peñarreta-Quezada et al., 2025). From a gender perspective, the pandemic intensified pre-existing inequalities: women, already performing three times more unpaid care work than men, often deprioritised their own health, a burden exacerbated in rural areas where care work intersects with physically demanding labour (Sami Gabriela Sánchez Verduga, 2025). Our analyses indicated that the inverse association between BDH-TP and maternal mortality was stronger during the COVID-19 pandemic than during the pre-/post-pandemic period, whereas no significant difference was observed for BDH-EP. This suggests that the broader population coverage measure may have captured programme effects that became more apparent under the exceptional social and economic conditions of the pandemic. Nevertheless, BDH alone was insufficient to prevent the overall increase in maternal mortality observed during this period. While global guidance identifies income transfer programmes as key instruments for crisis mitigation and social resilience (Cavalcanti et al., 2025), further evidence is needed to clarify BDH's potential to mitigate health shocks.

Similarly, the 2013-2014 update restricting BDH eligibility to households in extreme poverty was associated with a stronger inverse association between BDH-TP and maternal mortality, whereas no corresponding change was observed for BDH-EP. One possible explanation is that narrowing programme eligibility altered the geographical distribution of beneficiaries and the overall penetration of the programme within communities, which is more directly reflected by BDH-TP than by coverage among eligible households. However, these findings should be interpreted cautiously, as the ecological design does not allow the mechanisms underlying these differences to be established.

Our study is, to our knowledge, the first to examine the association between BDH and maternal mortality in Ecuador over a 14-year period, with multiple sensitivity analyses supporting the robustness of the findings. However, some limitations should be noted. First, our study uses an ecological design, which is susceptible to ecological fallacy, as BDH coverage is measured at the canton level and cannot capture individual or household-level exposure or mechanisms. However, canton-level analysis allows assessment of population-level effects, including potential spillover effect on non-beneficiaries, for example through changes in overall service use or care-seeking behaviour. While individual-level mechanisms cannot be examined due to data availability, this approach is appropriate for evaluating population-level effects of social policies.

Second, BDH coverage data were available only from 2010 onwards, precluding the assessment of short-term effects related to programme introduction or early expansion. However, evidence from other CCT evaluations suggests that effects on maternal health tend to emerge after sustained exposure, reflecting cumulative changes in healthcare utilisation, education, nutrition, and household decision-making (Glassman et al., 2013; Rasella et al., 2021). Accordingly, omission of early implementation years is unlikely to substantially bias estimates of longer-term associations with maternal mortality.

Third, residual confounding is possible. Fixed-effects models cannot account for time-varying unmeasured confounders, such as changes in local health-system capacity, health reforms, or concurrent social programmes. Although we adjusted for key demographic, socioeconomic, and health-system variables, annual socioeconomic indicators were estimated by interpolating census data. If socioeconomic changes were not fully captured by the interpolated measures, residual confounding would most likely attenuate estimated associations, as mortality declined during periods of declining BDH coverage, biasing results toward the null rather than inflating estimated programme effects. Additionally, analyses considering ethnicity and urbanicity should be interpreted with caution, as these variables were derived from the 2022 census and treated as time-invariant proxies across the 2010–2023 period. Although both represent structural characteristics that typically change slowly at the canton level, some temporal misclassification is possible, particularly in areas experiencing migration or urban expansion. However, sensitivity analysis using ethnicity data from the 2010 census yielded consistent findings (Table S8), suggesting that the results were robust to the choice of census year. Furthermore, although our classification does not capture within-canton ethnic diversity, it provides a reasonable proxy for the ethnic composition of cantons given the available data.

Finally, data quality poses additional challenges. Maternal deaths are recorded by place of occurrence rather than residence, likely underestimating mortality in rural, high-BDH cantons and overestimating it in better-resourced cantons receiving referrals, biasing estimates towards the null. Sensitivity analyses at the province level and restricted to cantons with hospital beds (Tables S6 and S7) yielded results consistent with the main analysis, suggesting that this non-differential misclassification did not materially affect the findings. To improve internal validity, cantons with low-quality vital statistics were excluded; although this may reduce generalisability, sensitivity analyses including all cantons and only excluded cantons (Tables S3 and S4) produced similar estimates, indicating limited selection bias. Maternal mortality is a rare event, and canton-level estimates, particularly in smaller population, are sensitive to small fluctuations, and data for 2020–2023 remain provisional (INEC, 2022), potentially affecting analyses related to the Covid-19 pandemic. To address potential instability, analyses were repeated restricting the sample to cantons with populations greater than 10,000, with no meaningful change in results (Table S5). Overall, sensitivity analyses suggest that the main conclusions are robust to data limitations, although some residual uncertainty remains. Improved maternal death registration, including recording by place of residence and standardised cause-of-death certification, is essential for accurate evaluation.

## 5 Conclusions

CCTPs have the potential to support maternal health in disadvantaged populations, particularly through demand-side mechanisms such as promoting antenatal care. In Ecuador, BDH effectively targeted structurally disadvantaged cantons, yet its association with maternal mortality was generally limited. Our findings contribute to the body of evidence that cash transfers alone are unlikely to substantially reduce maternal deaths. Continued progress will likely require sustained investment in both upstream social determinants of health, including poverty reduction and education, and equitable access to high-quality maternal healthcare.

Overall, BDH represents a valuable social protection tool and a platform for integrating maternal health interventions. The limited association observed in this study suggests that programme implementation, quality of care, and the monitoring of health-related conditionalities may influence the extent to which social protection translated into measurable improvements in maternal health. Further research should focus on identifying the pathways through which BDH may affect maternal health, particularly intermediate outcomes such as antenatal care and skilled birth attendance. Natural experiment designs could strengthen causal inference and help clarify programme mechanisms in the context of rare and multifactorial outcomes like maternal mortality.

## CRediT authorship contribution statement

**Sara Toros:** Writing – review & editing, Writing – original draft, Visualization, Software, Methodology, Investigation, Formal analysis, Data curation, Conceptualization. **Ronal Ruiz:** Writing – review & editing, Validation, Software, Methodology, Data curation, Conceptualization. **Samuel A.G. da Silva:** Writing – review & editing, Validation, Investigation, Data curation. **Pablo Álvarez:** Writing – review & editing, Validation, Software, Resources, Data curation. **Monsermin Gualán:** Writing – review & editing, Validation, Project administration, Investigation, Data curation. **Klauss K.S. Garcia:** Writing – review & editing, Validation, Investigation. **Mauricio L. Barreto:** Writing – review & editing, Validation, Project administration, Methodology, Funding acquisition. **Philip J. Cooper:** Writing – review & editing, Validation, Project administration, Investigation, Funding acquisition. **Peter Craig:** Writing – review & editing, Validation, Methodology, Investigation, Funding acquisition. **Natalia Romero-Sandoval:** Writing – review & editing, Validation, Project administration, Investigation, Funding acquisition, Conceptualization. **Julia M. Pescarini:** Writing – review & editing, Validation, Supervision, Project administration, Methodology, Investigation, Funding acquisition, Conceptualization. **Ana L. Moncayo:** Writing – review & editing, Validation, Supervision, Methodology, Investigation, Funding acquisition, Conceptualization.

## Ethics

This study was based on secondary analysis of publicly available data obtained from Ecuadorian national databases; as such, ethical approval and informed consent were waived by the London School of Hygiene & Tropical Medicine.

## Declaration of generative AI and AI-assisted technologies in the manuscript preparation process

During the preparation of this work the author(s) used ChatGPT to improve sentence structure and the readability of the manuscript. After using this tool/service, the author(s) reviewed and edited the content as needed and take(s) full responsibility for the content of the published article.

## Declaration of competing interest

The authors report no conflicts of interest.

## Data Availability

The data used in this study are publicly available from Ecuadorian government websites. The links available to download data were appropriately cited in the manuscript.
