# Supplementary material for: Long-term association of a conditional cash transfer programme and maternal mortality in Ecuador: An ecological longitudinal study, 2010-2023
Source: SSM Popul Health. 2026 Aug 31;35:101963. doi: 10.1016/j.ssmph.2026.101963 (PMC13587903; doi:10.1016/j.ssmph.2026.101963)
Supplement: Supplementary file 1 [file mmc1.docx]

**Supplementary Materials**

**Table of Contents**

| **Table** | **Content** |
| --- | --- |
| **Table S1** | TIDieR-PHP checklist |
| **Table S2** | Descriptive statistics of included and excluded cantons |
| **Table S3** | Analysis including all cantons |
| **Table S4** | Analysis including only low-quality vital statistics cantons |
| **Table S5** | Analysis restricted to cantons with >10,000 inhabitants |
| **Table S6** | Analysis restricted to cantons with hospital bed availability |
| **Table S7** | Province-level analysis |
| **Table S8** | Analysis using 2010 Census ethnicity classification |
| **Table S9** | Analyses using one-year lagged and three-year average BDH coverage |
| **Table S10** | Analysis using Poisson regression models |
| **Table S11** | Rate ratios per 10%-point increase in BDH coverage |
| **Table S12** | Interaction models with health-system characteristics |
| **Table S13** | Interaction models using the Ecuadorian Deprivation Index (EDI) |
| **Technical Appendix** | Selection of the interpolation method for the Unsatisfied Basic Needs Index (UBNI) poverty variable |

**Supplementary Table 1. TIDieR-PHP report (CEPAL, 2025; Martinez et al., 2017; MIES)**

| **1. Brief name** | Bono de Desarrollo Humano (BDH) – Conditional Cash Transfer Programme (CCT). |
| --- | --- |
| **2. Why** | The BDH is a CCT programme part of Ecuador’s non-contributory social safety net, within the National Social Inclusion and Equity System. Implemented by the Ministry of Economic and Social Inclusion (MIES), the programme aims to provide income support to low-income households with children under 18 years old.  The BDH seeks to alleviate poverty, promote human capital development, and guarantee a minimum level of consumption for families in extreme poverty, encouraging them to invest in the education and health of children from disadvantaged backgrounds. Conditionalities linked to health and education aim to have both short- and longer-term outcomes (e.g. children having better opportunities later in life). |
| **3. What materials** | Beneficiaries receive guidance on programme requirements and co-responsibilities through national communication campaigns on radio and television, used also to inform families about how to verify if they have been selected (call centre, Registro Social website, and local MIES service offices).  Nature and value of benefit: monthly cash transfers provided to eligible households.  Physical resources: no direct physical resources are provided; however, the programme aims to facilitate access to existing educational and healthcare services. |
| **4. What and how** | Funding: Inter-American Development Bank (IDB), International Bank for Reconstruction and Development (IBRD). Currently only the National Government.  Legal framework: Executive Decree No.347-A /2003; Executive Decree No.12 /2007; Executive Decree No.1824 /2006; Executive Decree No. 1838/2009; Executive Decree No. 129 of August 23, 2017.  Eligibility: households living in extreme poverty – implies that some households with no children or adolescents are also eligible to receive the BDH.  Selection process carried out in 2 phases:   1. The National Secretariat of Planning and Development (SENPLADES) identifies the poorest census sectors through information from the National Institute of Statistics and Censuses (INEC) and the Unsatisfied Basic Needs Index developed by the Andean Community of Nations. Based on this information, the census sectors where poverty incidence exceeds 50% of households are selected. For example, in 2013, 24,482 census sectors were identified using this criterion (country total of 42,649 census sectors). 2. Surveys of each household residing in these sectors to assess their economic situation and identify potential beneficiaries.   A demand-based registration procedure at MIES office allows households living in census sectors not identified as poor to request inclusion in the BDH.  Once the socioeconomic information is collected and integrated into the ‘Registro Social’, the ‘Indice de Bienestar’ is calculated for each family (combination of 34 survey variables, which include housing characteristics, access to services, asset ownership, household composition, and education levels, among others). Index values between 0-100, where 100 is the highest level of well-being - as of April 2015, the eligibility cutoff point was 28.20, designed to include extremely poor households, defined as those with a score equal to or below 24.07, along with a margin to reduce exclusion errors.  Registro Social sent to MIES, which selects beneficiaries, and their payments are activated. They are responsible for finding out if they were selected. The government provides several public information channels, including a call center, the Registro Social website, and local MIES service offices. The SENPLADES and MIES conduct mass communication campaigns on radio and television to inform families about how to verify if they have been selected.  Service delivered: monetary incentive is USD 50, paid monthly. In 2018, transfer was changed to be variable, up to a maximum of USD 150 per month depending on the number of minor children in the household (Executive Decree No. 99). No electronic card, or bank account or virtual wallet. Broad network of payment locations (>9,000), >20 financial institutions, including rural cooperatives. A central switch managed by MIES confirms beneficiary eligibility when IDs are presented at payment points, via an intermediary known as the ‘aggregator switch’. Financial institution delivers the transfer. Option to use ATMs, although for this purpose, beneficiaries must have the MIES Bono Rápido card.  Payment calendar: beneficiaries can collect their payment on days that match the last digit of their national ID number, or on weekends. Beneficiaries can accumulate up to 4 months of payments – but accounts reset in Jan each year.  Conditionalities: BDH beneficiary households are expected to meet basic co-responsibilities in education, health, and housing. Children aged 5–17 must attend school regularly, with a minimum attendance of 75%, and those under 15 are prohibited from working. In health, pregnant women must complete five prenatal visits, and children under one year are required to attend six check-ups. Children aged 1–5 must receive at least two medical visits per year. Adults of reproductive age are expected to attend an annual family planning session. In terms of housing, beneficiaries are instructed not to construct homes in high-risk areas such as flood zones or landslide-prone regions.  Sanctions: Non-compliance with co-responsibilities may lead to payment reductions or suspension. In education, failure to enrol a child results in a 50% reduction, while failure to meet attendance requirements leads to permanent suspension. In health, repeated non-compliance triggers progressive penalties, starting with a warning and escalating from a 20% reduction to full suspension after multiple breaches. However, in practice, there is no systematic process to monitor compliance with co-responsibilities, and no payment suspensions have been applied to beneficiaries who fail to meet them. |
| **5. Who provided** | Responsible organisation: MIES  Executing organisation: Undersecretariat of Non-Contributory Insurance, Contingencies and Operations attached to the Vice Ministry of Economic Inclusion of the MIES.  >20 financial institutions, including rural cooperatives, provide payments. 7 ‘aggregator switches’ check eligibility. |
| **6. Where** | Geographic scale: national. Community level, engaging with households directly.  Broad network of payment locations (>9,000).  Between 2001 and 2014, Ecuador, an upper middle-income country, experienced significant economic and social progress, with GDP growth averaging 4.5% annually, surpassing the regional average. This growth, along with improved income distribution, lifted 1.4 million people out of poverty. The most notable gains occurred between 2007 and 2014, when poverty and extreme poverty rates declined sharply, and the income of the poorest 40% grew faster than the national average. These improvements were largely driven by increases in labour income and government transfers, particularly in rural areas. Access to basic services also improved, with reductions in child mortality and expanded educational enrolment. However, starting in 2014, falling oil prices exposed fiscal vulnerabilities, limiting the government’s ability to sustain social programs. As public debt rose and growth declined, poverty reduction stalled, and labour market informality increased—especially among women, youth, and older workers. |
| **7. When and how often** | BDH implemented since 2003 and ongoing. Monetary incentive is paid monthly to beneficiaries. |
| **8.1 Planned variation** | The BDH was explicitly designed to be flexible over time, with planned adjustments in transfer amounts and co-responsibilities. For example, the cash transfer was increased several times to reflect inflation and household needs, and in 2018 it was redesigned to be variable, linked to the number of children. Similarly, co-responsibilities in education and health were progressively specified to align with national development goals. These planned changes were part of the programme’s strategy to improve targeting and strengthen its human capital objectives. |
| **8.2 Unplanned variation** | The process for identifying BDH beneficiaries (both definition of target population and selection mechanisms) has been modified in 2009 and in 2013. In 2014, 60% of BDH beneficiaries exited the programme (target population changed from those living in poverty to extreme poverty) – in April 2015, the number of beneficiary households had dropped to 440,000, a significant decrease from the 946,000 households registered in April 2014.  Co-responsibilities and consequences for no compliance were established in 2003 and revised in 2013 (stricter for health, education, new requirements related to housing conditions, employment, child begging). |
| **9.1 How well** | The BDH programme is managed through a standardized, centralized system administered by MIES. Eligibility, benefit activation, and updates are handled via the Registro Social, which integrated socioeconomic data nationwide. Payment distribution is managed through an “aggregator switch” system connected to a network of over 9,000 payment points and financial institutions, ensuring timely and uniform delivery across provinces. Mass media campaigns and local outreach also support clear communication of programme rules and conditionalities. |
| **9.2 How well—delivery** | In practice, the programme delivers cash transfers reliably through its network of payment channels. However, enforcement of conditionalities is weak or absent. Evaluations have shown that although co-responsibilities are clearly defined, there is minimal monitoring and sanctions are rarely applied, even in cases of non-compliance. This divergence between design and implementation limits the delivery fidelity of core components intended to drive health and education outcomes. |

# **Supplementary Table 2. Descriptive statistics of included and excluded cantons.**

| **Characteristic** | **Included** | **Excluded** |
| --- | --- | --- |
| N of cantons | 147 | 74 |
| Population size, median (IQR) | 30186 (15500 – 72955) | 14612 (7896 – 36262) |
|  |  |  |
| **Continuous variables, median (IQR)** |  |  |
| MMR | 20.2 (0.00 – 40.7) | 27.1 (0.00 – 59.9) |
| BDH TP | 24.4 (17.0 – 32.2) | 29.8 (22.7 – 37.2) |
| BDH EP | 42.7 (34.9 – 49.6) | 46.9 (39.8 – 53.8) |
| Physicians | 14.1 (9.80 – 20.1) | 15.8 (10.8 – 21.4) |
| Beds | 0.50 (0.00 – 1.00) | 0.00 (0.00 – 0.90) |
| Poverty | 57.7 (47.7 – 69.9) | 65.0 (56.0 – 77.7) |
| Illiteracy | 6.81 (4.62 – 9.51) | 6.42 (4.80 – 8.90) |
|  |  |  |
| **Dominant ethnicity, median (IQR)** |  |  |
| Mestizos | 86.5 (61.6 – 95.8) | 69.6 (40.4 – 93.6) |
| Afro-Ecuadorians | 1.11 (0.44 – 2.04) | 0.95 (0.30 – 2.95) |
| Indigenous | 1.23 (0.23 – 8.53) | 2.11 (0.35 – 42.9) |
| Montubios | 0.62 (0.31 – 6.54) | 0.41 (0.24 – 2.15) |
|  |  |  |
| **Urbanicity, median (IQR)** |  |  |
| Urban population proportion | 36.2 (24.1 – 57.8) | 36.8 (21.7 – 47.3) |

# **Supplementary Tables 3-10. Sensitivity and Robustness Analyses.**

**Table S3.** Univariate and adjusted analysis of the association between BDH and MMR, using the full dataset (including cantons with low-quality vital statistics, n = 221).

|  | RR crude (95% CI)* | p-value** | RR adjusted (95% CI)*** | p-value** |
| --- | --- | --- | --- | --- |
| **BDH coverage** |  |  |  |  |
| Total population | 1.00 (0.99 – 1.02) | 0.15 | 1.00 (0.98 – 1.01) | 0.74 |
| Eligible population | 1.00 (0.99 – 1.00) | 0.05 | 0.99 (0.98 – 1.00) | 0.31 |

**Table S4.** Univariate and adjusted analysis of the association between BDH and MMR, using the excluded canton dataset (only cantons with low-quality vital statistics, n = 74).

|  | RR crude (95% CI)* | p-value** | RR adjusted (95% CI)*** | p-value** |
| --- | --- | --- | --- | --- |
| **BDH coverage** |  |  |  |  |
| Total population | 1.02 (1.00 – 1.04) | <0.001 | 1.03 (1.01 – 1.05) | <0.01 |
| Eligible population | 1.01 (0.99 – 1.02) | <0.001 | 1.01 (1.00 – 1.03) | 0.11 |

**Table S5.** Univariate and adjusted analysis of the association between BDH and MMR, restricting the analyses to cantons with > 10.000 inhabitants.

|  | RR crude (95% CI)* | p-value** | RR adjusted (95% CI)*** | p-value** |
| --- | --- | --- | --- | --- |
| **BDH coverage** |  |  |  |  |
| Total population | 1.00 (0.98 – 1.01) | 0.30 | 0.99 (0.97 – 1.00) | 0.14 |
| Eligible population | 0.99 (0.98 – 1.00) | 0.01 | 0.99 (0.97 – 1.00) | 0.08 |

**Table S6.** Univariate and adjusted analysis of the association between BDH and MMR, restricting the analyses to cantons with availability of usable hospital beds.

|  | RR crude (95% CI)* | p-value** | RR adjusted (95% CI)*** | p-value** |
| --- | --- | --- | --- | --- |
| **BDH coverage** |  |  |  |  |
| Total population | 1.00 (0.98 – 1.02) | 0.30 | 0.99 (0.97 – 1.00) | 0.20 |
| Eligible population | 0.99 (0.98 – 1.00) | 0.02 | 0.99 (0.97 – 1.00) | 0.08 |

**Table S7.** Univariate and adjusted analysis of the association between BDH and MMR, using provinces as the unit of analyses. †

|  | RR crude (95% CI)* | p-value** | RR adjusted (95% CI)*** | p-value** |
| --- | --- | --- | --- | --- |
| **BDH coverage** |  |  |  |  |
| Total population | 1.00 (0.98 – 1.01) | 0.23 | 0.99 (0.97 – 1.00) | 0.05 |
| Eligible population | 0.99 (0.97 – 1.01) | 0.01 | 0.99 (0.98 – 1.00) | 0.07 |

† Poverty quintiles and illiteracy tertiles included as continuous variables due to sparse data and collinearity.

**Table S8.** Stratified associations between BDH coverage and MMR estimated from interaction models with ethnicity, derived from the 2010 National Census.

|  | **BDH-TP** | | **BDH-TP** |  |
| --- | --- | --- | --- | --- |
|  | RR (95% CI)*** | p-value** | RR (95% CI)*** | p-value** |
| **Ethnicity** |  |  |  |  |
| Mestizos | 0.99 (0.97 – 1.03) | 0.89 | 0.99 (0.98 – 1.01) | 0.43 |
| Afro-Ecuadorians | 0.97 (0.95 – 0.99) | < 0.001 | 0.99 (0.97 – 1.00) | 0.03 |
| Indigenous | 0.99 (0.95 – 0.99) | 0.56 | 0.99 (0.97 – 1.00) | 0.08 |
| Mixed | 0.98 (0.96 – 1.00) | 0.11 | 0.98 (0.97 – 1.00) | 0.06 |
| Montubios | 0.99 (0.97 – 1.01) | 0.44 | 0.99 (0.97 – 1.00) | 0.18 |

**Table S9.** Univariate and adjusted analysis of the association between BDH and MMR, using one-year lagged and three-year average BDH coverage.

|  | RR crude (95% CI)* | p-value** | RR adjusted (95% CI)*** | p-value** |
| --- | --- | --- | --- | --- |
| **BDH coverage** |  |  |  |  |
| BDH-TP (Lag 1) | 1.00 (0.99 – 1.01) | 0.32 | 0.99 (0.98 – 1.01) | 0.41 |
| BDH-TP (Avg 3) | 1.01 (0.99 – 1.03) | 0.28 | 1.00 (0.98 – 1.03) | 0.76 |
|  |  |  |  |  |
| BDH-EP (Lag 1) | 0.99 (0.97 – 1.00) | 0.02 | 0.98 (0.97 – 1.00) | 0.05 |
| BDH-EP (Avg 3) | 0.98 (0.96 – 1.00) | 0.26 | 0.98 (0.96 – 1.00) | 0.08 |

*Estimated using negative binomial regression for panel data with fixed effects.

**Estimated using the Wald test for association.
*** Estimated using negative binomial regression for panel data with fixed effects, and adjusted for poverty, illiteracy, physicians’ rate and hospital beds rate.

RR = Rate Ratio; CI = Confidence Interval; BDH-TP = coverage of total population; BDH-EP = coverage of eligible population; Lag 1 = 1-year lagged BDH coverage; Avg 3 = average BDH coverage over the previous three years (excluding current year).

**Table S10.** Univariate and adjusted analysis of the association between BDH and MMR, estimated using a Poisson model specification.

|  | RR crude (95% CI)* | p-value** | RR adjusted (95% CI)*** | p-value** |
| --- | --- | --- | --- | --- |
| **BDH coverage** |  |  |  |  |
| Total population | 1.00 (0.99 – 1.01) | 0.96 | 0.99 (0.97 – 1.00) | 0.09 |
| Eligible population | 0.99 (0.98 – 1.00) | 0.10 | 0.99 (0.97 – 1.00) | 0.03 |

*Estimated using Poisson regression for panel data with fixed effects.

**Estimated using the Wald test for association.
*** Estimated using Poisson regression for panel data with fixed effects, and adjusted for poverty, illiteracy, physicians’ rate and hospital beds rate.

RR = Rate Ratio; CI = Confidence Interval

**Supplementary Table 11. Adjusted rate ratios of the association between BDH and MMR, calculated for a 10%-point increase in BDH coverage.**

|  | **BDH-TP** | **BDH-EP** |
| --- | --- | --- |
|  | RR (95% CI)* | RR (95% CI)* |
| **Fully adjusted model** | 0.88 (0.74 – 1.05) | 0.88 (0.77 – 1.01) |
|  |  |  |
| **Poverty quintile (UBN Index)** |  |  |
| Q1 (Poorest) | 0.91 (0.73 – 1.15) | 0.94 (0.78 – 1.14) |
| Q2 | 1.03 (0.81 – 1.31) | 1.02 (0.86 – 1.21) |
| Q3 | 0.81 (0.60 – 1.07) | 0.87 (0.72 – 1.05) |
| Q4 | 0.66 (0.50 – 0.87) | 0.81 (0.70 – 0.93) |
| Q5 (Richest) | 0.69 (0.46 – 1.03) | 0.90 (0.78 – 1.03) |
|  |  |  |
| **Ethnicity** |  |  |
| Mestizos | 0.93 (0.73 – 1.18) | 0.91 (0.78 – 1.05) |
| Afro-Ecuadorians | 0.77 (0.64 – 0.93) | 0.88 (0.78 – 0.99) |
| Indigenous | 0.96 (0.78 – 1.19) | 0.90 (0.77 – 1.04) |
| Mixed | 0.81 (0.62 – 1.06) | 0.88 (0.75 – 1.03) |
| Montubios | 0.93 (0.77 – 1.13) | 0.92 (0.79 – 1.06) |
|  |  |  |
| **Urbanicity** |  |  |
| Rural | 1.00 (0.83 – 1.21) | 0.96 (0.82 – 1.13) |
| Intermediate | 0.84 (0.67 – 1.07) | 0.88 (0.75 – 1.03) |
| Urban | 0.77 (0.61 – 0.97) | 0.88 (0.76 – 1.02) |

* Estimated using negative binomial regression for panel data with fixed effects, and adjusted for poverty, illiteracy, physicians’ rate and hospital beds rate. Reported rate ratios and 95% confidence intervals correspond to a 10-percentage-point increase in BDH coverage.

RR = Rate Ratio; CI = Confidence Interval; BDH-TP = coverage of total population; BDH-EP = coverage of eligible population; UBN = Unsatisfied Basic Needs.

**Supplementary Table 12. Stratified associations between BDH coverage and MMR estimated from interaction models with health-system characteristics.**

|  | **BDH-TP** | | **BDH-EP** | |
| --- | --- | --- | --- | --- |
|  | RR (95% CI)* | p-value** | RR (95% CI)* | p-value** |
| **Physicians’ density** |  |  |  |  |
| Low | 1.00 (0.98 – 1.02) | 0.05 | 0.99 (0.98 – 1.01) | 0.84 |
| Intermediate | 0.98 (0.96 – 1.00) |  | 0.99 (0.98 – 1.00) |  |
| High | 0.97 (0.94 – 1.00) |  | 0.99 (0.97 – 1.00) |  |
|  |  |  |  |  |
| **Hospital beds availability** |  |  |  |  |
| Low | 0.97 (0.94 – 1.00) | <0.01 | 0.97 (0.95 – 0.99) | 0.02 |
| Intermediate | 1.00 (0.98 – 1.02) |  | 1.00 (0.98 – 1.02) |  |
| High | 0.98 (0.95 – 1.00) |  | 0.99 (0.97 – 1.00) |  |

*Estimated using negative binomial regression for panel data with fixed effects and adjusted for relevant socioeconomic and health-system variables.

**Wald test for the interaction between BDH coverage and health-system variable.

RR = Rate Ratio; CI = Confidence Interval; BDH-TP = coverage of total population; BDH-EP = coverage of eligible population.

**Supplementary Table 13. Stratified associations between BDH coverage and MMR estimated from interaction models with Ecuadorian Deprivation Index (EDI).**

|  | **BDH-TP** | | **BDH-EP** | |
| --- | --- | --- | --- | --- |
|  | RR (95% CI)* | p-value** | RR (95% CI)* | p-value** |
| **EDI Quintile** |  |  |  |  |
| Q1 (Lowest) | 0.88 (0.81 – 0.96) | <0.001 | 0.99 (0.97 – 1.00) | 0.32 |
| Q2 | 0.93 (0.90 – 0.96) |  | 0.98 (0.97 – 1.00) |  |
| Q3 | 0.97 (0.94 – 0.99) |  | 0.99 (0.98 – 1.00) |  |
| Q4 | 0.97 (0.95 – 1.00) |  | 0.99 (0.97 – 1.00) |  |
| Q5 (Highest) | 0.99 (0.97 – 1.01) |  | 1.00 (0.98 – 1.01) |  |

*Estimated using negative binomial regression for panel data with fixed effects and adjusted for relevant socioeconomic and health-system variables.

**Wald test for the interaction between BDH coverage and EDI.

RR = Rate Ratio; CI = Confidence Interval; BDH-TP = coverage of total population; BDH-EP = coverage of eligible population.

**TECHNICAL APPENDIX**

**Selection of the Interpolation Method for the Pobreza NBI Variable**

*at the Canton Level in Ecuador (2010–2022)*

**1. Introduction**

In the econometric model presented in this article, the **NBI** index is included as a covariate at the canton level. In Ecuador, NBI data at this geographic disaggregation are available exclusively for the census years **2010 and 2022**, as produced by the National Institute of Statistics and Censuses (INEC). To construct a complete annual series covering the 2010–2022 period required for the panel data estimation, an interpolation procedure was applied to generate values for the intermediate years.

This appendix documents the methodological rationale for selecting **linear interpolation** over an **exponential decay** specification. The comparison is grounded in a trend-consistency criterion: the cantonal averages of each interpolated series are contrasted against the annual national NBI poverty rate derived from the National Survey of Employment, Unemployment and Informality (*ENEMDU*), which provides yearly estimates of poverty incidence at the national level.

**2. Interpolation Methods**

**2.1 Linear Interpolation**

Linear interpolation assumes a constant rate of change between the two census anchor points (2010 and 2022). For canton *i* in year *t*, the estimated value is given by:

*NBI_i(t) = NBI_i(2010) + [(NBI_i(2022)−NBI_i(2010))/12]×(t − 2010)*

This specification distributes the observed inter-census change uniformly across all intermediate years, yielding an annual decrement of approximately 1.77 percentage points in the cantonal average.

**2.2 Exponential Decay Interpolation**

The exponential decay specification assumes a declining rate of change over time, implying that poverty reduction is most rapid at the beginning of the period and decelerates progressively toward the terminal year. The functional form adopted is:

*NBI_i(t) = NBI_i(2022) + [NBI_i(2010)− NBI_i(2022)]×e^(−λ(t − 2010))*

where *λ* is a decay parameter calibrated such that, the function passes exactly through both census endpoints. Under this specification, the steepest annual reduction occurs in the initial year of the interpolation window.

**3. Validation Criterion**

Given the absence of direct cantonal NBI observations for the intermediate years, validation relies on a **trend-consistency criterion**. For each interpolation method, the simple average of cantonal interpolated rates was computed for each year from 2010 to 2022 and compared with the **annual national NBI poverty rate reported by the ENEMDU** over the same period.

This approach is justified on the grounds that, although the two data sources measure conceptually related but methodologically distinct constructs—ENEMDU provides annual household survey estimates with national representativeness, whereas census-based NBI reflects a multidimensional deprivation index—both capture the *direction and pace of poverty change* at the national level. Consequently, the interpolation method whose temporal trajectory most closely mirrors that of the ENEMDU series offers the most plausible approximation to the underlying dynamics of the variable.

Crucially, the criterion focuses on **dynamic consistency**—that is, the year-on-year direction and magnitude of change—rather than on the coincidence of absolute levels, since the latter is inherently constrained by the methodological differences between the two sources.

**4. Results**

Table A1. National ENEMDU poverty rate and cantonal interpolated averages, 2010–2022

| **Year** | **ENEMDU (%)** | **Linear Interpolation (%)** | **Exp. Decay Interpolation (%)** |
| --- | --- | --- | --- |
| 2010 | 41.81 | 76.23 | 76.23 |
| 2011 | 39.42 | 74.46 | 70.20 |
| 2012 | 36.77 | 72.68 | 68.66 |
| 2013 | 38.68 | 70.91 | 67.18 |
| 2014 | 35.42 | 69.14 | 65.74 |
| 2015 | 32.85 | 67.36 | 64.36 |
| 2016 | 32.01 | 65.59 | 63.02 |
| 2017 | 31.81 | 63.82 | 61.73 |
| 2018 | 33.54 | 62.04 | 60.49 |
| 2019 | 34.16 | 60.27 | 59.28 |
| 2020 | 33.20 | 58.50 | 58.12 |
| 2021 | 33.15 | 56.72 | 56.99 |
| 2022 | 31.44 | 54.95 | 54.95 |

*Note: ENEMDU = National Survey of Employment, Unemployment and Informality (INEC). Linear and Exp. Decay columns report the simple average of cantonal-level interpolated NBI rates. Anchor years (2010, 2022) are identical across both methods by construction.*

**4.1 Trend Analysis**

The linear interpolation produces a steady annual reduction of **−1.77 percentage points** in the cantonal average, a trajectory that closely mirrors the gradual and sustained decline observed in the ENEMDU series, which records annual changes ranging from −0.20 to −3.26 percentage points over the period, with two minor reversals in 2013 and 2018–2019 attributable to cyclical economic factors.

By contrast, the exponential decay specification generates an initial drop of **−6.02 percentage points** between 2010 and 2011, followed by a progressive flattening to decrements of approximately 1.1–1.5 percentage points per year in the latter part of the period. This pattern implies that poverty reduction was disproportionately concentrated in the first year of the interpolation window—a dynamic that **finds no empirical support** in the national trend, which exhibits a comparatively smooth and continuous decline throughout the entire period.

**4.2 Figure**

*Figure A1. Comparison of interpolated cantonal UBN averages with the ENEMDU national poverty rate, 2010–2022. Note: Vertical axis scales are not directly comparable across series due to methodological differences between the UBN census-based index and the ENEMDU income-based poverty measure.*

**4.3 Implications for Econometric Estimation**

The artificial concentration of change produced by the exponential decay specification would introduce *temporal heterogeneity* in the covariate that is not grounded in observed data, potentially inducing spurious correlations between the NBI variable and other time-varying regressors in the model. In particular, the steep initial decline followed by stagnation could distort the estimated coefficient on NBI by confounding genuine structural effects with the artifact of the interpolation procedure.

The linear specification, by distributing the inter-census change uniformly, avoids the introduction of such systematic distortions and is consistent with the principle of *minimal parametric assumptions* in the treatment of missing observations.

**5. Conclusion**

Based on the trend-consistency analysis documented above, **linear interpolation** was selected as the method for generating the annual cantonal NBI series for the 2010–2022 period. This decision rests on three methodological grounds:

**(1) Trend consistency.** The cantonal average of the linear interpolation replicates the pattern of gradual and sustained poverty reduction observed in the national ENEMDU series more faithfully than the exponential decay alternative.

**(2) Parsimony and absence of unverifiable assumptions.** Linear interpolation does not require the estimation of a decay parameter λ, thereby avoiding the risk of introducing additional bias through calculated parameters.

**(3) Minimization of temporal distortions.** By avoiding the artificial concentration of change in specific years, linear interpolation reduces the risk of inducing heteroscedasticity or spurious correlations in the panel estimation.

It is acknowledged that this interpolation strategy constitutes a simplifying assumption inherent to the structure of the available data. The limitation is reported transparently as part of the methodological considerations of the article. This approach is consistent with practices documented in the empirical literature on multidimensional poverty in Latin America, where linear interpolation between census waves is a standard procedure in the absence of higher-frequency sub-national data (see, e.g., Alkire & Foster, 2011; CEPAL, 2018).

**References**

Alkire, S., & Foster, J. (2011). Counting and multidimensional poverty measurement. *Journal of Public Economics*, 95(7–8), 476–487.

CEPAL. (2018). *Panorama Social de América Latina 2018*. Comisión Económica para América Latina y el Caribe.

INEC. (2010, 2022). *Censo de Población y Vivienda*. Instituto Nacional de Estadística y Censos del Ecuador.

INEC. (2010–2022). *Encuesta Nacional de Empleo, Desempleo y Subempleo (ENEMDU)*. Instituto Nacional de Estadística y Censos del Ecuador.
